# RMP/URI inhibits both intrinsic and extrinsic apoptosis through different signaling pathways

**DOI:** 10.7150/ijbs.36829

**Published:** 2019-10-15

**Authors:** Yuan Ji, Jian Shen, Min Li, Xiaoxiao Zhu, Yanyan Wang, Jiazheng Ding, Shunyao Jiang, Linqi Chen, Wenxiang Wei

**Affiliations:** 1 Department of Cell Biology, Institute of Bioengineering, School of Medicine, Soochow University, Suzhou 215123, China; 2 Department of Interventional Radiology, First Affiliated Hospital of Soochow University, Suzhou, 215006, China; 3 Department of Tumor, People Hospital of Maanshan, Maanshan, 243000, China; 4 Department of Endocrinology, Children's Hospital affiliated to Soochow University, Suzhou, 215000, China

**Keywords:** RMP, Apoptosis, ATM, NF-κB, P53

## Abstract

The evading apoptosis of tumor cells may result in chemotherapy resistance. Therefore, investigating what molecular events contribute to drug-induced apoptosis, and how tumors evade apoptotic death, provides a paradigm to explain the relationship between cancer genetics and treatment sensitivity. In this study, we focused on the role of RMP/URI both in cisplatin-induced endogenous apoptosis and in TRAIL-induced exogenous apoptosis in HCC cells. Although flow cytometric analysis indicated that RMP overexpression reduced the apoptosis rate of HCC cells treated with both cisplatin and TRAIL, there was a difference in mechanism between the two treatments. Western blot showed that in intrinsic apoptosis induced by cisplatin, the overexpression of RMP promoted the Bcl-xl expression both *in vitro* and *in vivo*. Besides, RMP activated NF-κB/p65(rel) through the phosphorylation of ATM. However, in TRAIL-induced extrinsic apoptosis, RMP significantly suppressed the transcription and expression of P53. Moreover, the forced expression of P53 could offset this inhibitory effect. In conclusion, we presumed that RMP inhibited both intrinsic and extrinsic apoptosis through different signaling pathways. NF-κB was distinctively involved in the RMP circumvention of intrinsic apoptosis, but not in the extrinsic apoptosis of HCC cells. RMP might play an important role in defects of apoptosis, hence the chemotherapeutic resistance in hepatocellular carcinoma. These studies are promising to shed light on a more rational approach to clinical anticancer drug design and therapy.

## Introduction

Hepatocellular carcinoma (HCC) is the third leading cause of cancer-related mortality and the main cause of death in cirrhotic patients worldwide 1. Systematic chemotherapy has been one of the most important strategies applied to HCC treatment in addition to surgical resection 2. However, the various chemotherapeutic drugs currently in use against HCC show a strong chemoresistance, which exerts a massive hindrance on therapeutic agents targeting HCC 3. Cisplatin (CDDP) and TNF Related Apoptosis Inducing Ligand (TRAIL) are representative anticancer agents applied to the standard regimens of various cancers in the genitourinary, respiratory, and digestive organs 4. However, most HCC patients in chemotherapy prove tolerant to CDDP and TRAIL though the molecular mechanism remains unclear 5. It has been reported that the response rate of HCC patients to CDDP given as systemic monotherapy only 15% 4. Subsequently, investigating the molecular mechanism underlying chemoresistance and developing new therapeutic targets become urgent to improve the effects of chemotherapy in HCC patients.

A poor response to chemotherapeutic regimens in the treatment of liver cancer might attribute to complex mechanisms such as the activation of cell survival signaling, the translocation of telomerase into mitochondria, tumor microenvironment formation and the most common apoptosis resistance 6,7. Many studies have emphasized the fact that the impairment of apoptotic machinery mainly contributes to the resistance to chemotherapeutic regimens including TRAIL and CDDP 4,5. Furthermore, it has been reported that the chemoresistance of HCC cells is attributed to the evasion from apoptosis to a large extent 8. Thus, it becomes especially noteworthy to focus on the deregulation of signaling pathways governing cell apoptosis to overcome the resistance to chemotherapeutic agents. As is known to all, there are two different signaling pathways to induce apoptosis: the intrinsic and the extrinsic signaling pathway respectively 9. The point of no return in the intrinsic apoptotic pathway involves mitochondrial outer membrane permeabilization (MOMP) by pro-death proteins, while the extrinsic apoptotic pathway involves the binding of death ligands of the tumor necrosis factor (TNF) family to their specific death receptors (DRs) on cell surface 10,11. In particular, TRAIL and CDDP could induce the extrinsic and the intrinsic apoptotic pathway respectively. Thus, from a mechanistic perspective, it is vital to find new molecular target regulating these two critical apoptotic pathways above in order to overcome the drug resistance in HCC cells.

RNA polymerase II subunit 5 (RPB5)-mediating protein (RMP/URI), also known as unconventional prefoldsin RPB5 interactor (URI), was first isolated and cloned from a human HepG2 cDNA library more than a decade ago. Recently, several studies have reported that RMP is an oncogene that plays a vital role in tumor proliferation, invasion and metastasis 12. A previous study revealed that the overexpression of RMP enhances the resistance of cervical cancer cells to CDDP, although the concrete mechanism remains unclear 13. Besides, we have previously shown that RMP inhibited the intrinsic apoptotic pathway induced by irradiation. However, there is still no evidence that RMP plays an important role in extrinsic apoptotic pathway. Based on the evidence above, we suspect that RMP might play a vital role in the deregulation of these two critical apoptotic pathways considering that HCC is resistant to CDDP and TRAIL treatment.

In this study, we report for the first time the inhibitory effect of RMP on both intrinsic and extrinsic apoptotic pathways induced by CDDP and TRAIL respectively. Notably, it has been reported that HCC cells are resistant to TRAIL-induced apoptosis, possibly due to several distinct mechanisms, including the compensatory activation of AKT and NF-κB. Unexpectedly, we did not find the activation of NF-κB induced by RMP overexpression, but a significant repression on P53 expression upon TRAIL treatment. It indicated that RMP might inhibit extrinsic apoptosis through a different pathway, in which P53 was involved. Moreover, the recovery expression of P53 abolished this inhibitory effect by RMP, which further supports the role of P53 inhibition in the RMP-mediated resistance of TRAIL in HCC cells. On the other hand, RMP inhibited intrinsic apoptotic pathway through NF-κB activation, accompanied by the parallel increase in the expression of NF-κB target gene products including Bcl-xL. However, the activation of ATM instead of AKT was involved in this process. Our data indicated that RMP inhibited intrinsic and extrinsic apoptosis through different signaling pathways. The inhibitory role of RMP on two critical apoptotic pathways explains the chemoresistance of HCC to TRAIL and CDDP treatment from a mechanistic perspective. RMP might serve as a promising target which could increase the sensitivity of HCC patients towards chemotherapeutic agents clinically.

## Materials and methods

### Plasmids, Cell Lines and Reagents

The following cells were used in this study: HepG2, stable cell lines PCDNA3.1-RMP/URI-HepG2, pGPU6-RMPi-HepG2 were saved in our lab. The plasmid pGPU6-Neo was purchased from Jima Co (Shanghai, China). The plasmid pGPU6/Neo-RMPi for RMP depletion and pCDNA3.1-RMP/URI for RMP over expression were constructed as described previously 14. The Annexin-V/PE apoptosis detection kit was purchased from BD Biosciences (Shanghai, China).

GeneTran^TM^ III was obtained from Invitrogen and Qiagen. Protein A/G PLUS-Agarose was purchased from the Santa Cruz Biotechnology. Halt Protease, Phosphatase Inhibitor Cocktail and EDTA-Free were obtained from Thermo and BeyoECL Plus from Beyotime. Protein A/G agarose beads (Sc-2003, Santa Cruz). The following antibodies were used in this study: 93H1 (anti-Phospho-NF-κB/p65(rel) or P65(Ser536), Cell Signaling Technology); Sc-8392(anti-Bcl-xl, Santa Cruz Biotechnology); Sc-83129 (anti-AKT1/2/3, Santa Cruz Biotechnology); Sc-7985-R (anti-p-AKT1/2/3(Ser473), Santa Cruz Biotechnology); Sc-371 (anti-IκB-α, Santa Cruz Biotechnology); Sc-8404 (anti-p-IκB-α, Santa Cruz Biotechnology); Sc-376011 (anti-RMP/URI, Santa Cruz Biotechnology); ab81292 (anti-phospho-ATM(Ser1981, abcam); ab78 (anti-ATM, abcam); ab7970 (anti-NF-κB/P65(rel), abcam); Sc-2027 (normal rabbit IgG, Santa Cruz Biotechnology); Sc-2025 (normal mouse IgG, Santa Cruz Biotechnology); Goat Anti-Rabbit IgG(H+L)-HRP and Rabbit Anti-Mouse IgG(H+L)-HRP (Good Science); Sc-126 (anti-P53(DO-1), Santa Cruz Biotechnology).

### Cell culture and transfection

Cells were cultured in Dulbecco's modified Eagle's medium (DMEM) supplemented with 10% fetal bovine serum (FBS) (Sino-American Biotechnology Co, Shanghai, China). Before transfection, 4×10^5^ cells were plated into each well of the 6-well plate. After incubating for 24h, these cells were transfected with GeneTran^TM^III according to the manufacturer's instructions. Cells were transfected with plasmids pGPU6/Neo-RMPi for RMP depletion (RMPi) and pCDNA3.1-RMP/URI for RMP overexpression (RMPo). Then transfected cells were cultured at 37℃ in a humidified incubator containing 5% CO2.

### Detection of apoptosis

Cells were stained with Annexin-V/PE apoptosis detection kit according to the manufacturer's instructions. Briefly, cells were washed for two times with cold PBS and then suspended with 1×Binding Buffer in a concentration of 1x10^6^cells/ml. From which 100ul suspensions were taken out and stained with 5ul PE Annexin-V plus 5ul 7-AAD for 15min at 25℃ in the dark, followed by 400ul 1×Binding Buffer adding to each tube. Finally cells were analyzed by flow cytometry.

### Measurement of mitochondrial membrane potential (Δψm)

Cells stained with JC-1 were applied for flow cytometry to detect mitochondrial membrane potential (Δψm). Then cells were treated with cisplatin (12μg/ml) (not treated as control) for 48h and stained with JC-1 for 30m in culture medium at 37℃.Washed with PBS, cells were resuspended and analyzed immediately using flow cytometer. Red fluorescence was measured in the FL2 mode and green fluorescence measured in the FL1 mode. Mitochondrial depolarization was indicated by a decrease in the red/green fluorescence intensity ratio. For each sample, 10000 events were acquired.

### RT-PCR

RT-PCR was carried out as described previously 15. The sequences of primers involved in PCR reaction were as followed: Pro-Caspase8 (Forward: 5'-GAG CTG CTC TTC CGA ATT-3', reverse, 5'-TCC AAC TTT CCT TCT CCC-3'), DR4 (Forward: 5'-AAG TCC CTG CAC CAC GAC-3', reverse, 5'-CCA CAA CCT GAG CCG ATG), GAPDH (Forward: GAC CTG ACC TGC CGT CTA-3', reverse, 5'-AGG AGT GGG TGT CGC TGT-3'). P53 (Forward: 5'-CCG TCT GGG CTT CTT GCA TT-3', reverse, 5'-CGC CTC ACA ACC TCC GTC AT-3').

### Western blot analysis

Cells were washed with PBS and harvested with lysis buffer. Separation of protein bands was subjected to 10% sodium dodecyl sulfate-polyacrylamide gel electrophoresis (SDS-PAGE), and electro-transferred to a nitrocellulose membrane. The membrane was then blocked with 5% BSA in Tris Buffered Saline (pH 7.5) with 0.1% Tween 20. The membranes were then incubated overnight with the PARP-1(1:1000) and caspase-3 (1:1000) antibody, which were used as a marker for cell death. Other antibodies used in this study include NF-κB/p65(rel) (1:1000), Bcl-xl (1:500), IκBa(1:500) and phospho-NF-κB/p65(1:1000), AKT(1:1000), P-AKT(1:1000), ATM(1:2000), P-ATM(1:5000), P53 (1:1000) proteins were detected by incubation with their respective primary antibodies. Rabbit Tubulin antibody was used as control. Membranes were washed 3 times with PBS-Tween (0.2% Tween-20) for 10 min each, followed by incubation with horse-radish peroxidase-conjugated secondary antibody for 2 hours at room temperature. The immune complexes were visualized by the ECL chemiluminescent method using Clarity Western ECL Substrate (Bio-Rad, California, USA).

### Co-immunoprecipitation (Co-IP)

Co-IP experiments were performed essentially as described previously 16. Briefly, for conventional IP experiments, cells were cultured in 6cm cell culture dishes to 90% confluence. The cells were then either harvested without additional treatments or after treatment with DDP. Cells were then lysed with IP buffer (50mM Tris-HCl, pH 7.4, with 150mM NaCl, 1mM EDTA and 1%TritonX-100) in the presence of a protease inhibitor mixture. Protein were precleared using 60 mL of Protein A/G agarose beads and incubated with 5ul RMP antibody and 20ul of protein A/G-agarose bead suspension, rotation for a night at 4℃.After 3 washes with extraction buffer, the immunoprecipitation proteins were dissolved in 20 mL of laemmli buffer, boiled for 5 min at 99℃, and analyzed on 10% SDS-PAGE and transferred to nitrocellulose membrane for Western blot analysis.

### Nude Mice and Human Tissue Samples

For the *in vivo* tumor formation, the nude mice were divided into 3 groups, each consisting of 5 mice. 5×10^6^ HepG2 cells with RMP overexpression (RMPo) and depletion (RMPi) in 0.1 ml of PBS were respectively injected subcutaneously into the right flank of 15-20g female nude mice (Animal Centre of Soochow University). Xenograft tumors developed in the nude mice six weeks later after injection and then the mice tumors were dissociated for Immunohistochemistry. Hepatocellular carcinoma and the matched non-tumor hepatic tissues were obtained from The Third Affiliated Hospital of Soochow University.

### Immunohistochemistry Detection

Immunohistochemistry was performed as the instructions of Biotin-Streptavidin HRP Detection Systems. Briefly, the tissue sections were dewaxed, rehydrated and then immersed in methanol containing 0.3% hydrogen peroxide for 30 min to block endogenous peroxidase activity then washed 3 times in PBS (3 minutes at RT). The slides were blocked in 1% blocking serum for 30 min then incubated in the primary polyclonal antibodies against RMP(1:200), Bcl-xl(1:200), phospho-NF-κB/p65 (1:100) and P-ATM(1:70)overnight then washed 3 times in PBS (3 minutes at RT).Incubated with biotinylated goat anti-mouse IgG for 15 min then washed 3 times in PBS (3 minutes at RT).The sections were then incubated with DAB for 10 min for visualization of the peroxidase reaction.

## Results

### RMP inhibited the cisplatin-induced endogenous apoptosis in HCC cells

**FIGURE 1:**
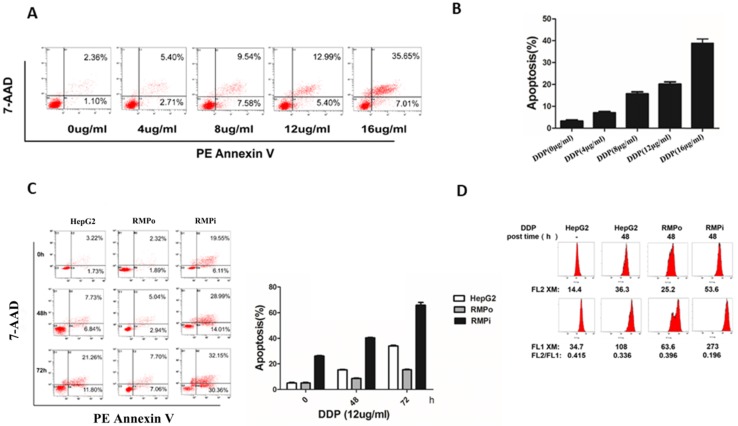
RMP inhibited the cisplatin-induced endogenous apoptosis of HCC cells. **(A)** HepG2 cells were treated with different concentrations of cisplatin (0, 4, 8, 12 and 16ug/ml) for 48h, and then cells were harvested and apoptosis analysis was analyzed by flow cytometry. **(B)** The percentage of apoptotic cells was scored and depicted graphically. Cisplatin enhanced the apoptosis rate of HepG2 cells in a dose-dependent manner. **(C)** HepG2 and two stable cell lines PCDNA3.1-RMPo-HepG2 (RMPo), pGPU6-RMPi-HepG2 (RMPi) were treated with cisplatin (12μg/ml) for the indicated time and the cells were harvested to be subjected to flow cytometry. **(D)** Cells were stained with JC-1 and the mitochondrial electrochemical potential gradient was analyzed by flow cytometry. The decline of red/green fluorescence intensity ratio represents mitochondrial depolarization, which indirectly reflects the occurrence of endogenous apoptosis. This ratio was much lower in RMPi HepG2 groups than in control after cisplatin treatment, whereas it increased slightly once RMP was over-expressed.

Our previous work demonstrated that RMP is a cellular oncogene playing an important part in genotoxic stress (60Co-irradiation)-induced apoptosis 17,18. However, whether RMP plays a similar inhibitory role in apoptosis induced by chemotherapeutic agents still remains unclear. To investigate the role of RMP in chemotherapeutic agents-induced apoptosis, cisplatin was used as an apoptosis inducer, which could promote the apoptosis of hepatocellular carcinoma cells. HepG2 cells were treated with the increasing concentration of cisplatin (0, 4, 8, 12 or 16ug/ml) for 48h and then subjected to apoptotic analysis with flow cytometry analysis. Results showed that cisplatin increased the apoptosis rate of HepG2 in a dose-dependent manner (Figure 1A&B). As the apoptosis rate started reaching a proper range in the concentration of 12μg/ml (the apoptotic rate was 18.39%), it was chosen as the working concentration in the following experiment.

To examine the effect of RMP on cisplatin-induced apoptosis in HCC, we established the stable expression (RMP overexpression, RMPo) or interference of RMP (RMP interference, RMPi) in HepG2 cell line. Then HepG2, RMPo and RMPi HepG2 cell lines were treated with 12μg/ml cisplatin for three different time points: 0h, 48h and 72h and the results were shown in Figure 1C.Although the apoptosis rate in all three groups got higher with the increasing time course after the incubation with cisplatin, a striking depletion of RMP resulted in a higher apoptosis rate in these groups than in control groups. Since HepG2 cells steadily overexpressing RMP showed a lower apoptotic rate compared with that of control group, they seemed to be more resistant to cisplatin treatment. These results above demonstrate that RMP could inhibit the cisplatin-induced apoptosis in HCC cells.

Mitochondrial depolarization was an important process and indicator in the mitochondrial-mediated endogenous caspase apoptosis pathway. To determine the effect of RMP on mitochondrial depolarization, HCC cells were treated with cisplatin (12μg/ml) and stained with JC-1 and their mitochondrial membrane potential was measured by flow cytometry. Mitochondrial depolarization was indicated by a decrease in the red/green fluorescence intensity ratio. As shown in Figure 1D, the mitochondrial membrane potential of HepG2 cells before treatment was 0.451. The mitochondrial membrane potentials in all groups treated with cisplatin declined, but the intensity ratio of FL2/FL1 in RMPo cells (0.396) was slightly higher than that of HepG2 cells (0.336), whereas the ratio of FL2/FL1 in RMPi cells (0.196) was significantly lower than that of HepG2 cells (0.336). It indicated that RMP might prevent mitochondrial from depolarization, which in turn inhibits the endogenous apoptosis of HCC cells. Nevertheless, the depletion of RMP renders mitochondria more sensitive to cisplatin, resulting in the decrease of mitochondrial membrane potential. These results suggested that RMP could inhibit cisplatin-induced endogenous apoptosis through up-regulating the Bcl-xl expression in HepG2 cells.

### RMP promoted the protein expression of Bcl-xl, but suppressed the cleavage of PARP

**FIGURE 2:**
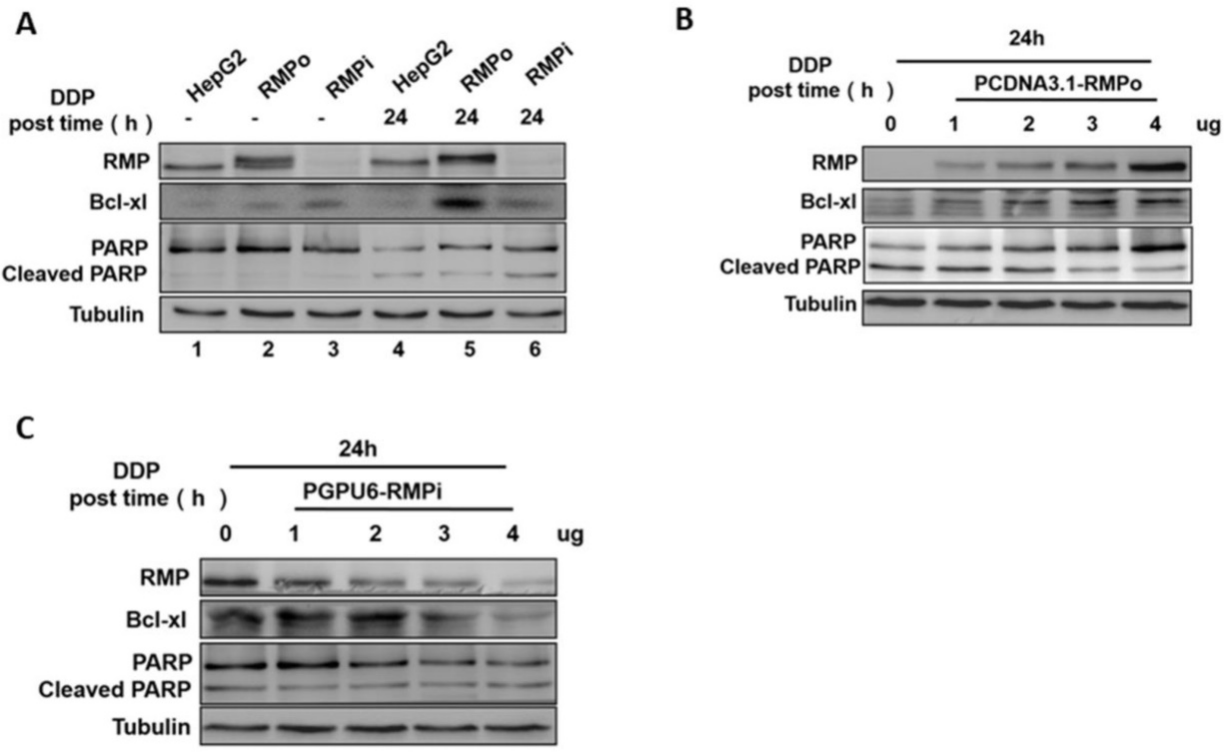
RMP promoted the expression of Bcl-xl, but suppressed the cleavage of PARP. **(A)** After 24h treatment with cisplatin (12μg/ml), proteins were extracted and prepared for western blot analysis. The expression of Bcl-xl was significantly elevated in RMP over-expressing groups compared with control (Lane 5), and the cleavage of PARP obviously improved in RMPi HepG2 (Lane 6). **(B)** HepG2 cells were transfected with an increasing amount of PCDNA3.1-RMP-HepG2 (RMPo) (0, 1, 2, 3 or 4μg) and then treated with cisplatin for 24h. Total protein extracts were applied for Western blot. RMP overexpression enhanced the expression of Bcl-xl and repressed the cleavage of PARP in a dose-dependent manner. **(C)** HepG2 cells were transfected with pGPU6-RMPi-HepG2 (RMPi) and western blot analysis was carried out.

Previous studies have reported that Bcl-2 protein family member Bcl-xl could hinder the activity of mitochondrial permeability transition channel and protect it from mitochondrial outer membrane permeabilization (MOMP) and programmed cell death (PCD) 19,20. MOMP is a central apoptotic event primarily controlled by the Bcl-2 family proteins including Bcl-xl. There have been reports that Bcl-xl is associated with the multidrug resistance of tumors 21. PARP (Poly(ADP-ribose)polymerase) is one of the main cleavage targets of caspase-3, and the PARP cleavage is regarded as an important marker of apoptosis 22, 23. We examined the effect of RMP on the expressions of Bcl-xl and PARP cleavages in HCC cells after cisplatin treatment. Western blot analysis demonstrated that the expression of Bcl-xl was significantly elevated in RMP overexpressing groups compared with control (Figure 2A Lane 5). However, a lower PARP cleavage was observed in RMPo groups than in control (Figure 2A Lane 6). There was no significant difference in the expression of Bcl-xl and PARP cleavage among the three groups without cisplatin treatment (Figure 2A). This observation was confirmed further by transfecting HepG2 cells with an increasing amount of vectors. PCDNA3.1-RMPo vectors were applied to RMP overexpression (Figure 2B) and pGPU6/Neo-RMPi for RMP depletion (Figure 2C). As shown in Figure 2B, the expression of Bcl-xl was enhanced with increasing vector concentrations in a dose-dependent manner in RMPo groups, whereas the level of cleaved PARP was reduced. However, in RMPi groups, the situation was opposite (Figure 2C), which was consistent with the results in Figure 2A and Figure 2B.

### RMP inhibited the endogenous apoptosis of HepG2 cells through the NF-κB signaling pathway in HCC cells

**FIGURE 3:**
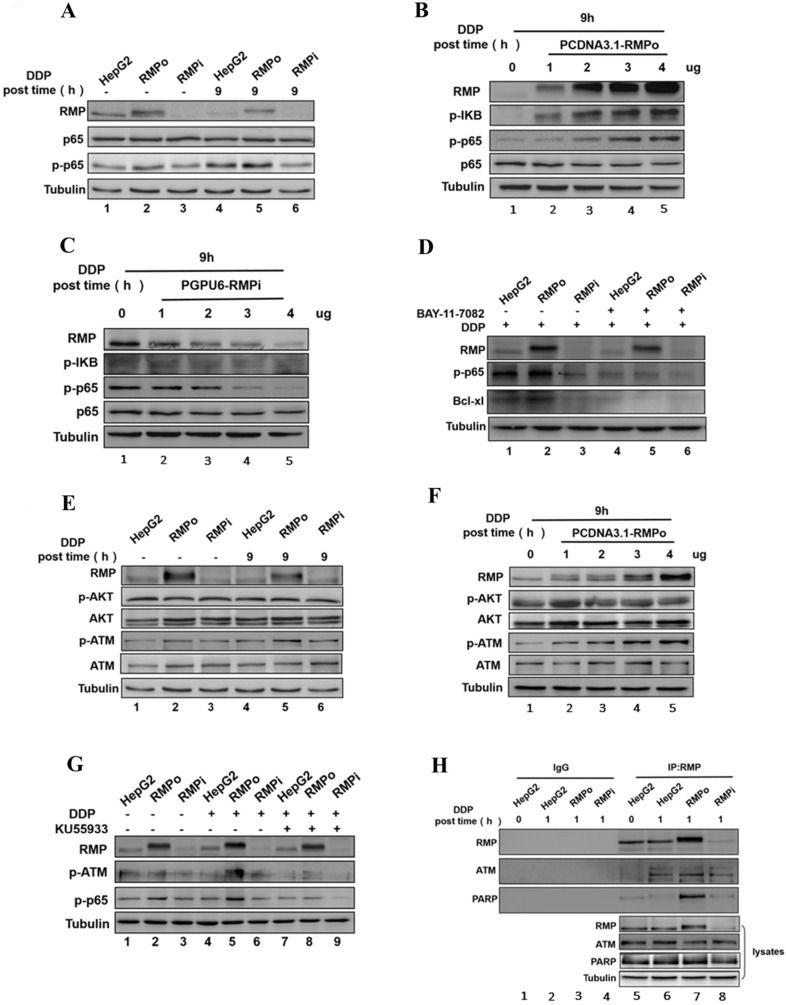
RMP inhibited the endogenous apoptosis of HCC cells through NF-κB signaling pathway. **(A)** HepG2, RMPo and RMPi cells were treated with cisplatin (12μg/ml) for 9h and lysates were prepared for western blot. Results indicated the phosphorylation of P65 apparently increased in RMPo HepG2 cells, but decreased after RMP interference. **(B)** HepG2 cells were transfected with an increasing amount of PCDNA3.1-RMPo vectors (RMPo) (0, 1, 2, 3 or 4μg) and the protein extracts were prepared for western blot analysis. RMP enhanced the phosphorylation of P65 and IΚB in a dose-dependent manner. **(C)** On the contrary, the phosphorylation of P65 gradually decreased with an increasing amount of PCDNA3.1-RMPo vectors (RMPi). **(D)** HepG2 cells were treated with bay-11-7082, an inhibitor of p65 phosphorylation, for 2h and the protein extracts were applied for western blot. RMP promoted the phosphorylation of ATM necessary for the activation of p65 and endogenous apoptosis pathway inhibition. **(E)** HepG2, RMPo and RMPi cells were treated with cisplatin (12μg/ml) for 9h and lysates were prepared for western blot. Phosphorylation of ATM significantly increased in RMP-overexpressing HepG2 cells. **(F)** RMP enhanced the phosphorylation of ATM in a dose-dependent manner. HepG2 cells were transfected with an increasing amount of PCDNA3.1-RMPo plasmids (RMPo) (0, 1, 2, 3 or 4μg) and cell lysate were used for western blot. **(G)** After incubation with KU55933, a specific inhibitor of ATM kinase, cells were collected and applied for western blot analysis. Treated with cisplatin, the phosphorylation of ATM declined significantly in RMP over-expressing HepG2 cells after incubation with KU55933. **(H)** Co-immunoprecipitation showed an interaction among RMP, ATM and PARP and this interaction was enhanced after RMP overexpression.

The nuclear transcription factor-NF-κB has been reported to play an important role in controlling the transcription and expression of many anti-apoptotic proteins including Bcl-xl, which further influence cell apoptosis 24. Considering that Bcl-xl is a potential target gene of NF-κB, we examined if RMP could activate NF-κB signaling pathway in endogenous apoptosis induced by cisplatin in HCC cells. Western blot analysis showed that, whether treated with cisplatin or not, the overexpression of RMP (RMPo) promoted the phosphorylation of p65, but by contrast the total expression of p65 remained unchanged. Besides, the level of p65 phosphorylation significantly increased when cells were treated with cisplatin compared with untreated groups (Figure 3A Lane 5). To further confirm the activation of p65 by RMP, HepG2 cells were respectively transfected with an increasing amount of pCDNA3.1-RMPo (Figure 3B) and pGPU6/Neo-RMPi vectors (Figure 3C). Western blot analysis showed that the levels of the phosphorylated form of IκB and p65 were significantly enhanced with the increasing transfecting amount of RMPo vectors, although the total expression of p65 remained unchanged (Figure 3B). The situation was opposite with the depletion of RMP (Figure 3C). These results confirmed the activation of NF-κB by RMP.

It was previously reported that activated nuclear factor kappaB could translocate into nucleus and bind to Bcl-xl gene promoter region, promoting the transcription of targeted gene 25, 26. To investigate the role of NF-κB activation in transcription and expression of Bcl-xl, RMPo and RMPi HepG2 cell lines were pretreated with Bay-11-7082, an inhibitor of p65 phosphorylation, and then incubated with cisplatin. Western blot analysis, as shown in Figure 3D, demonstrated the protein levels of p-p65 and Bcl-xl in RMP stably expressing cells dramatically increased compared with groups untransfected with or depleted of RMP (Figure 3D Lane 2). After pretreatment with Bay-11-7082, however, the phosphorylation of p65 was almost eliminated. Meanwhile, the protein level of Bcl-xl also decreased in HepG2 cells with the overexpression of RMP (Figure 3DLane 5). These results indicated that Bcl-xl might be the downstream targeted gene of NF-κB and RMP promoted the expression of Bcl-xl depending on the activation of P65.

### RMP promoted the phosphorylation of ATM, which was necessary for both the activation of p65 and the inhibition of endogenous apoptosis pathway

As the PI3K/AKT/NF-κB signaling pathway is critical for the survival or apoptosis in cisplatin-based chemotherapy in cancers and NF-κB is one of downstream targets of AKT, we assume that AKT activation might play an essential role in RMP mediated NF-κB activation. After treatment with cisplatin for the indicated time, we examined the phosphorylation of AKT in RMPo and RMPi HepG2 cells by western blot analysis (Figure 3E). Actually, both the total expression of AKT and the phosphorylation level of AKT remain constant whether in HepG2 cells stably overexpressing either an RMP vector, a siRNA against RMP or an empty vector. Besides, the treatment with cisplatin did not affect the total or the phosphorylation level of AKT.

ATM is a nuclear protein kinase that regulates apoptosis and cell cycle checkpoint response after DNA double-strand breaks (DSBs). Previous study demonstrated that constitutively active ATM accounts for the activation of NF-κB 27, 28. Therefore, we tried to investigate the role of RMP in ATM activation induced by cisplatin targeted DNA damage. As shown in Figure 3E, without cisplatin incubation the overexpression of RMP in HepG2 cells resulted in a slight increase in the phosphorylation of ATM. It achieved a significant rise once RMPo HepG2 cells were treated with cisplatin (Figure 3E, Lanes 2 and 5), but the overall expression of ATM was not affected.

The above results showed that RMP did not affect the phosphorylation of AKT but activated the phosphorylation of ATM (Figure 3E). To further confirm the result, dose-dependent assay was performed. HepG2 cells were transfected with an increasing amount of pCDNA3.1-RMPo vectors followed by cisplatin treatment. Western blot analysis further confirmed that the total and phosphorylation protein level of AKT still remained constant with the increasing transfected RMP overexpression vectors. Interestingly, the phosphorylation of ATM significantly enhanced due to the increased expression of RMP, although the total ATM protein remained unchanged (Figure 3F).

To further confirm the conclusion that RMP did activate the phosphorylation of ATM, RMPo and RMPi HepG2 cells were pretreated with KU55933, an inhibitor of ATM phosphorylation, and then incubated with cisplatin. Western blot analysis demonstrated that after cisplatin treatment, the phosphorylation level of p65 and ATM in RMPo HepG2 groups (Lane 5) significantly increased than before (Lane 2). More importantly, after cisplatin treatment the overexpression of RMP dramatically increased the phosphorylation of both p65 and ATM (Lane 5) compared with HepG2 (Lane 4) and RMPi HepG2 groups (Lane 6) (Figure 3G). However, once KU55933 was applied, the elevation of ATM phosphorylation in RMPo cells was eliminated despite cisplatin treatment. Meanwhile, the phosphorylation of p65 also decreased in RMPo cells, which was similar to the level of RMPi cells. These results suggested that RMP activating NF-κB signal pathway was dependent on the activation of ATM.

As further proof, co-immunoprecipitation was carried out to examine the interaction between RMP and ATM (Figure 3H). HepG2 cells with RMP overexpression (RMPo) or knockdown (RMPi) were treated by cisplatin and subjected to apoptosis, followed by Co-IP. Results indicated that ATM and RMP were respectively co-precipitated with each other in HepG2 cells treated with cisplatin (Figure 3HLanes 6-8). In contrast, RMP and ATM were not precipitated by control antibody (Lane 2-4). Besides, more ATM protein was precipitated in HepG2 cells overexpressing RMP compared with untransfected cells, whereas little amount of ATM was pulled down from cells with RMP deletion. We concluded that RMP and ATM interacted with each other to form a protein complex.

The nuclear poly (ADP-Ribose)-dependent signalosome consisting of PARP, IKKa and PIASy contributes to the survival of injured proliferating cells and PARP, which is recognized as one of the important indicators to monitor apoptosis, is specifically cleaved during apoptosis 29. Considering this, we next examined the interaction between RMP and PARP. As shown in Figure 3H, PARP was also precipitated with RMP in cells with RMP overexpression, whereas very little amount of PARP was pulled down in RMPi cells. It suggested that RMP allied with both ATM and PARP to form a protein complex so as to resist the apoptosis in HCC cells.

### RMP promoted the expression of Bcl-xl and activation of ATM and p65 *in vivo*

**FIGURE 4:**
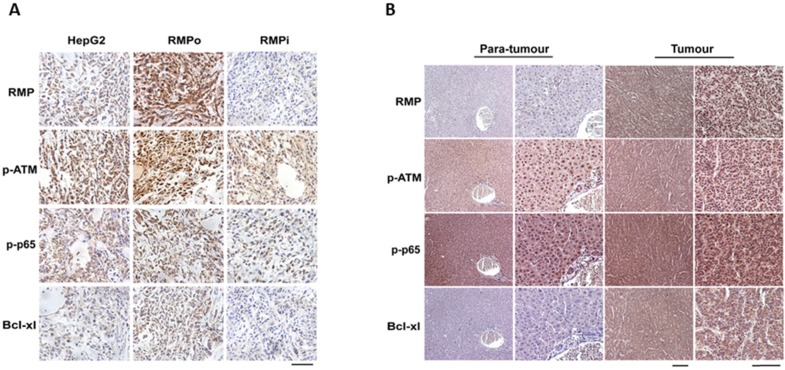
RMP promoted the expression of Bcl-xl and activation of ATM and p65 in hepatocellular carcinoma tissues. **(A)** Stably over-expressing RMP (RMPo) or depleted of RMP (RMPi) HepG2 cells were respectively inoculated subcutaneously into the dorsal flanks of nude mice. Two weeks later, tumors were sectioned and the expressions of RMP, p-ATM, p-p65 and Bcl-xl were detected by immunohistochemistry, Bar=100μm. **(B)** HCC tissues from patients and the matched non-tumor hepatic tissues adjacent to carcinoma from same patients were sectioned and applied for immunohistochemistry. Low magnification: Bar=200μm; high magnification: Bar=100μm.

To confirm the effect of RMP on the expressions of p-ATM, p-p65 and Bcl-xl, we further examined the expression levels of these proteins in tumor tissues from nude mice with hepatoma. As shown in Figure 4A, the expression of RMP increased in RMPo tumor tissues, but decreased once RMP was depleted. The phosphorylation of ATM and p65 both increased in HCC xenograft tumors implanted with RMPo HepG2 cells compared with tumors injected with HepG2 cells (HepG2) and RMP interference cells (RMPi). Similarly, there was an increase in the expression of anti-apoptotic factor Bcl-xl in tumor tissues overexpressing RMP, but a decrease in tissues depleted of RMP.

In addition, we also examined the effect of RMP on the expressions of p-ATM, p-p65 and Bcl-xl in HCC tissues and paired normal hepatic tissues (adjacent to carcinoma from the same patient) (Figure 4B). Immunohistochemistry results demonstrated that the expression of RMP in HCCs was significantly higher than that of paired normal hepatic tissues. Meanwhile p-ATM, p-p65 and Bcl-xl also showed an elevated expression in HCCs compared with non-tumor hepatic tissues, which is consistent with *in vitro* experiments.

### RMP inhibited the TRAIL-induced exogenous apoptosis pathway of HCC cells

**FIGURE 5:**
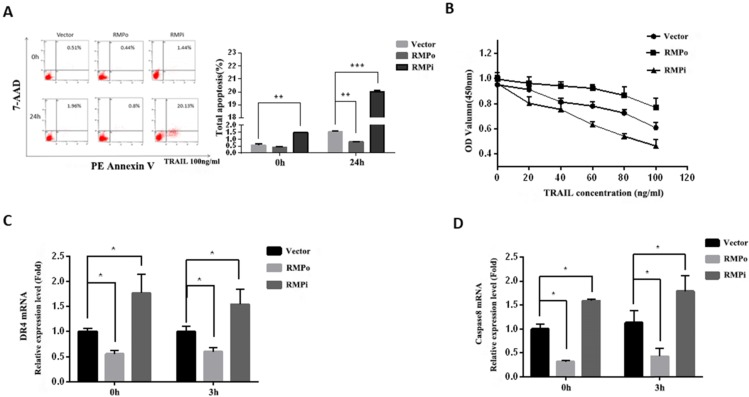
RMP inhibited the TRAIL-induced exogenous apoptosis pathway of HCC cells. **(A)** Stably over-expressing or depleted of RMP HepG2 cells were treated with 100ng/ml TRAIL for 24h. Then cells were harvested and applied for flow cytometric analysis. Results showed that RMP silencing significantly raised the apoptosis rate of HepG2 cells compared with empty control vector, whereas the apoptosis rate decreased in RMP over-expressing HepG2 cells.** (B)** After incubation with increasing concentrations of TRAIL (0, 20, 40, 60, 80, 100ng/ml), cell viability was analyzed by CCK-8 analysis. With the increasing concentration of TRAIL, there exhibited a gradual decline of OD values. These values increased in RMP over-expressing HepG2 cells, but decreased in RMP depleted cells. (C&D) HepG2 cells that stably over-expressing or depleted of RMP were treated with 100ng/ml TRAIL for 3h, total mRNA was extracted and real-time RT-PCR was used to assess the relative mRNA expression of DR4 **(C)** and caspase8 **(D)** respectively.

The results above have indicated that RMP could inhibit endogenous apoptosis induced by cisplatin, and then we tried to investigate the role of RMP in TRAIL-induced exogenous apoptosis pathway. After 24h incubation with TRAIL, RMPo, RMPi and control cells were harvested and analyzed by flow cytometry. Results showed that the total apoptosis rate of HepG2 cells remarkably increased after TRAIL incubation. There was a slight decline of apoptosis rate in RMPo cells as compared with control, whereas the apoptosis rate of RMPi cells increased by nearly 12 folds than that in the empty vector transfected HepG2 cells after TRAIL incubation (Figure 5A).

Further, we examined the dose-response relationship between cell proliferation and TRAIL concentration. CCK8 analysis showed that the OD value reduced with increasing TRAIL concentration; moreover, the OD value was even lower in RMPi cells than in cells transfected with empty vector (vector). However, the OD value of RMPo cells was higher than that of control (Figure 5B). This not only confirmed the cytotoxicity effect of TRAIL on tumor cells, but also suggested that RMP inhibited this effect of TRAIL, thus protecting HCCs from apoptosis.

TRAIL's pro-apoptotic function in humans is mediated by two closely related death-domain receptors, DR4 (TRAIL-R1) and DR5 (TRAIL-R2), which combine to contribute to exogenous apoptosis. As a biomarker of extrinsic apoptosis pathway, caspase-8 can activate downstream effector caspases independent of mitochondria once it is stimulated. In this study, we examined the expressions of DR4 and caspase-8 by RT-qPCR, the results of which were shown as Figure 5C and D respectively. After 3h incubation with TRAIL, no significant difference was observed between DR4 (Figure 5C) and caspase-8 (Figure 5D) mRNA expression after treatment. However, their expression in RMPo cells was lower than that in vector cells, with or without TRAIL treatment. Reverse effect was observed in RMPi cells. These results showed RMP suppressed the transcriptions of DR4 and caspase8, which indicated that RMP inhibited the extrinsic apoptosis pathway induced by TRAIL in HCC.

### NF-κB was not involved in the TRAIL-induced exogenous apoptosis suppressed by RMP

**FIGURE 6:**
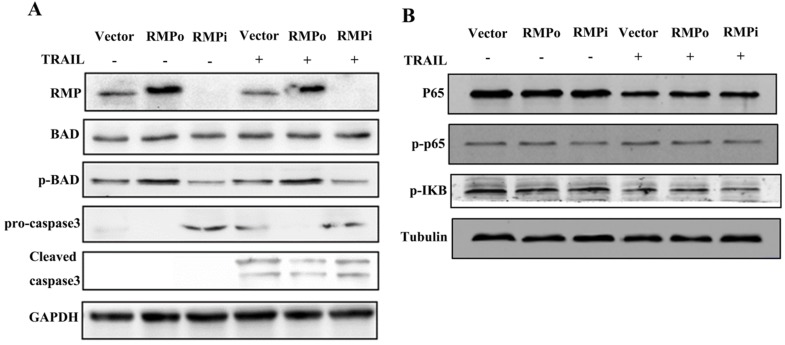
NF-κB was not involved in TRAIL-induced exogenous apoptosis pathway suppressed by RMP. **(A)** HepG2 cells, stably over-expressing RMP or depleted of RMP HepG2 cells were treated with 100ng/ml TRAIL and cell extracts were applied for western blot. RMP overexpression apparently enhanced the phosphorylation of BAD and reduced the cleavage of caspase3 slightly compared with vector control, whereas the situation was opposite in RMP silencing HepG2 cells (Lane 6). **(B)** Cells were treated as before, Western bolt analysis showed no significant change of p-p65 and p-IΚB expression in HepG2 after RMP over-expressing or silencing.

Immunoblot assay was performed to further confirm the inhibition of extrinsic apoptosis pathway induced by TRAIL. As shown in Figure 6A, the expression of p-BAD was enhanced significantly in RMP over-expressing cells than in vector control before and after TRAIL treatment, but declined in cells depleted of RMP. The expression of BAD in all groups, nevertheless, remained constant. A significant increase of the cleavage of caspase was observed after TRAIL treatment. Moreover, the pro-caspase expression and caspase3 cleavage in RMP over-expressing cells both decreased slightly after TRAIL incubation as compared with vector control. However, the expression of both pro-caspase and caspase3 increased in RMPi cells.

As is shown in Figure 3, RMP inhibited the endogenous apoptosis of HepG2 cells through NF-κB signaling pathway in HepG2 cells. We also examine whether the effect of RMP on the exogenous apoptosis induced by TRAIL was dependent on NF-κB. We next test the expressions of P65, p-p65 and p-IΚB in HepG2 cells (Figure 6B). Results have shown that unexpectedly no significant difference was observed among RMPo, RMPi and vector cells. These results suggested that unlike endogenous apoptosis, RMP inhibited exogenous apoptosis not through NF-κB signal pathway.

### RMP inhibited the expression of p53 in apoptosis extrinsic pathway in HCC cells and the forced expression of P53 could offset this inhibition effect

**FIGURE 7:**
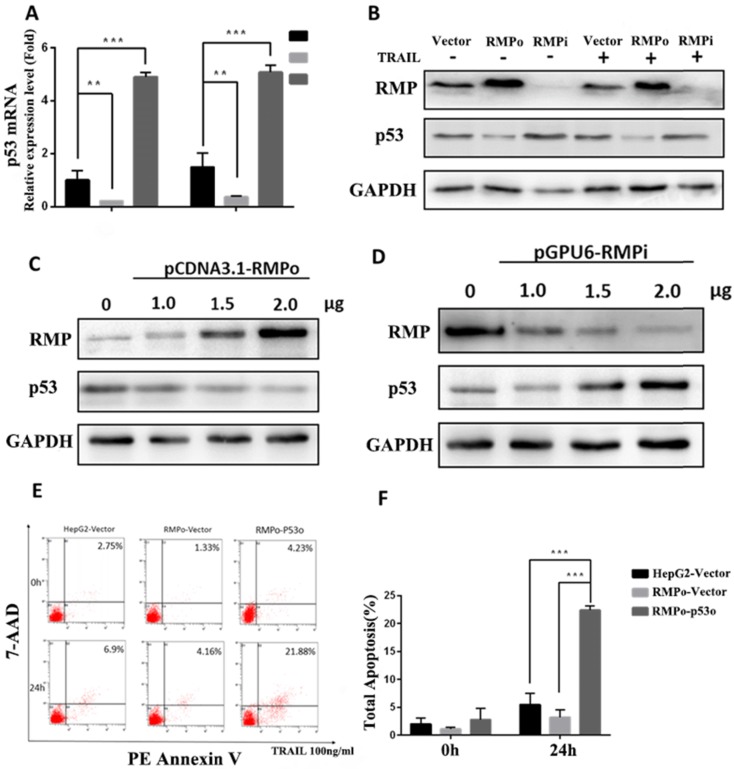
RMP repressed extrinsic apoptosis through the inhibition of P53 expression in HCC cells. **(A)**Stably over-expressing or depleted of RMP HepG2 cells were treated with 100ng/ml TRAIL for 3h and Real-time RT-PCR was applied for the detection of P53 relative mRNA expression. **(B)** Cells were treated as before and cell extracts were applied for western blot. Results indicated that RMP suppressed the protein expression of P53. **(C and D)** HepG2 cells were transfected with different concentrations of pCDNA3.1-RMPo and p-GPU6-RMPi plasmids and applied for western blot. Results indicated that RMP repressed the expression of P53 in a dose-dependent manner. **(E and F)** After 24h TRAIL treatment, cells were collected and the apoptosis rate was analyzed using flow cytometric assay. Results showed a significant increase of apoptosis rate after P53 overexpression in RMPo HepG2 cells.

Recent studies have reported that blocking p53 family function leads to chemoresistance in HCC. Besides, the stimulation experiment of CD95-, TNF- and TRAIL-receptor systems revealed that cytotoxic drugs could trigger the expression of each of these death receptors and consequently sensitize HCC cells toward apoptosis with the p53 family members as transactivators 30, 31. Therefore, we suspected whether RMP alleviated the sensitivity of HCC to TRAIL-induced extrinsic apoptosis pathway through P53 family function. RT-qPCR and Western blot analysis were respectively performed to investigate the effect of RMP on P53 gene transcription and protein expression (Figure 7A and B). As shown in Figure 7A, the P53 mRNA expression of RMPo cells reduced obviously compared with vector cells before and after TRAIL treatment. Whereas, the P53 mRNA expression in RMPi cells was a least 2 times higher than that of vector cells after TRAIL treatment. Meanwhile, a significant decline of P53 protein expression was observed in RMPo cells along with elevated P53 in RMPi cells compared with vector control before and after TRAIL treatment (Figure 7B).

To confirm the inhibitory effect of RMP on P53 expression, we further investigated the dose enhancement effects of RMPo and RMPi transfected vectors on the expression of P53. As shown in Figure 7C and D, there was a gradual decline of P53 protein with an increasing concentration of pCDNA3.1-RMPo plasmids and a rise of P53 with an increasing concentration of p-GPU6-RMPi plasmids, which suggested that RMP suppressed the expression of P53 in a dose-dependent manner.

**FIGURE 8:**
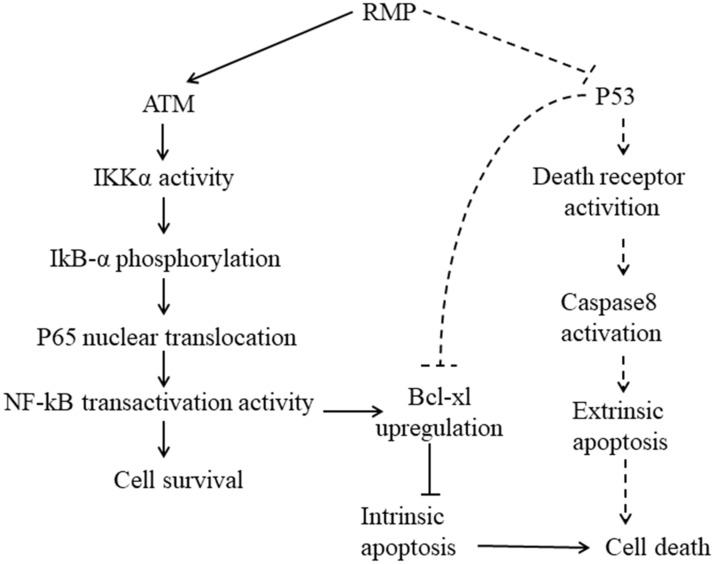
Working model. Solid lines: intrinsic apoptosis. Dashed lines: extrinsic apoptosis. When hepatoma carcinoma cells were treated with cisplatin, ATM was activated and combined with RMP, activating p65 to translocate into nucleus and promote the transcription of Bcl-xl. RMP tipped the balance of the ratio of pro-apoptotic and anti-apoptotic proteins and ultimately suppressed the intrinsic apoptosis response of HCC cells toward chemotherapy. However, the RMP repression of the extrinsic apoptosis induced by TRAIL was not dependent on NF-κB activation in which P53 played an important role. RMP triggered the expression of death receptors and consequently sensitized HCC cells toward extrinsic apoptosis via the p53 family members as transactivators. Besides, P53, as an important transcription factor, could also directly regulate the expression of Bcl-xl and finally control the intrinsic apoptosis. It should be noted that the intrinsic and extrinsic apoptosis are not entirely separable and these two pathways of apoptosis usually coordinated together to contribute to the regulation of apoptosis.

To further investigate whether RMP inhibited extrinsic apoptosis pathway induced by TRAIL via the suppression of P53 expression, we forced the expression of P53 in RMPo groups and tested the apoptosis rate of HepG2 cells before or after TRAIL treatment. As shown in Figure 7E and F, after TRAIL treatment, there was a decline of the apoptosis rate in RMPo groups. However, once the expression of P53 was forced in RMPo groups, the apoptosis rate increased by at least seven times and was even higher than in empty vector HepG2 cells. Our data showed that the forced expression of P53 could offset the inhibitory effect on exogenous apoptosis pathway. It suggested that in extrinsic pathway RMP suppressed apoptosis depending on P53 function instead of the activation of NF-κB, which was distinguished from intrinsic pathway (Figure 8).

## Discussion

A previous report revealed that the depletion of RMP increases CDDP and rapamycin-induced apoptosis in ovarian cancer cells by activating mitochondrial S6K1-BAD signaling pathway, which indicated that RMP might play an important role in the deregulation of apoptotic pathway 32. Based on this study, we further focused on the effect of RMP on intrinsic apoptosis induced by CDDP in HCC cells. Our data showed that the overexpression of RMP led to a decline in CDDP-induced apoptosis in HCC cells, while the apoptotic rate in the knock-down of RMP groups had more than tripled after 48 hours of CDDP treatment (Figure 1C). In most cases, mitochondrial outer membrane permeabilization (MOMP) defines the mitochondrial or intrinsic pathway of apoptosis and ultimately leads to caspase activation and protein substrate cleavage 33,34. In the present study, a JC-1 fluorescent probe detected the alteration of MOMP and flow cytometric analysis showed a significant decline of fluorescence intensity ratio (F2/F1) in HepG2 cells depleted of RMP, which indicated the activation of mitochondrial apoptotic pathway (Figure 1D). It suggested to us that HCC was intrinsically resistant to mitochondrial apoptotic pathway with the benefit of endogenous expression of RMP protein, which gave an innovative explanation for the mechanism of chemoresistance to CDDP in HCC. Our data further supported the result of LEE and his colleagues that the depletion of RMP activated the mitochondrial apoptotic pathway in HCC cells 32.

Another major finding in our study was that the overexpression of RMP significantly led to an enhanced expression of BCL-xl and reduced the cleavage of PARP (Figure 2). As a member of Bcl-2 family, Bcl-xl is generally considered to be a negative regulator of death executioner caspase-3 activation 35,36. The increased expression of BCL-xl hinders the relocalization of cytochrome c from mitochondria to the cytoplasm, where it stimulates the proteolytic processing of caspase precursors and caspase3 amplification activation, thus leading to the failure of down-stream cleavage of PARP and an impaired apoptosis process 37,38. Interestingly, the knock-down of RMP in our study did not significantly reduce the expression of Bcl-xl as expected. Considering the research findings of LEE that the depletion of RMP increases CDDP-induced apoptosis in ovarian cancer cells by activating mitochondrial S6K1-BAD signaling pathway 32, we suspect that the knock-down of RMP in HCC might probably involve the activation of pro-apoptotic family members such as BAD/BAX. Taken together, RMP is able to suppress mitochondrial permeability transition channel activity and block MOMP in response to CDDP stimuli through the up-regulation of BCL-xl, failed amplification of caspase activation and PARP cleavage, thus leading to impaired mitochondrial apoptotic mechanism.

NF-κB is an important transcription factor and has been shown to promote multi-gene expression including Bcl-xl 39,40. As an important phosphorylation site of NF-κB/P65, Ser536 is involved in regulation of transcriptional activity, nuclear localization and protein stability. Phosphorylation on Ser536 of NF-κB P65 has been shown to inhibit apoptosis in colon, breast and prostate and enhance tumor growth 41. The activation of NF-κB, depending on the phosphorylation and degradation of cytoplasmic inhibitor collectively termed IκB, was reported to inhibit the apoptosis in eosinophils after stress through promoting the transcription of its anti-apoptotic target gene Bcl-xl 42,43. However, the role of NF-κB in the inhibition of intrinsic apoptotic pathway of HCC is still poorly understood 44. In the present study, we showed that the overexpression of RMP increased the phosphorylation of NF-κB/p65 (rel) and IκB in HepG2 cells in a concentration-dependent manner (Figure 3A-C). Besides, this promoting effect of RMP was eliminated after its exposure to NF-κB inhibitor, indicating that the up-regulation of Bcl-xl by RMP and subsequent inhibition of apoptosis was largely dependent on NF-κB activation (Figure 3D). Remarkably, a previous study revealed that RMP mediated the chemoresistance of multiple myeloma through NF-κB/IL-6/STAT3 signaling pathway 45. In addition, the knockdown expression of RPB5, one of the polII subunits that provide a major anchoring site for other factors, led to a weak interaction between RMP and p65 46. The evidence suggests to us that RMP might be a regulator of NF-κB mediating the chemoresistance and intrinsic mitochondrial apoptosis in HCC, although the concrete mechanism is unclear and needs further study.

Autophosphorylation on Ser1981 of ATM and the ensuing transition from an inactive dimer to an active monomer represents a major activation process in our understanding. It has been recognized autophosphorylation of ATM at Ser1981 is a sign of DNA double-strand breaks and is indispensable for a proper DNA damage response, which further inhibit intrinsic apoptosis 47. In the other hand, full activation of AKT requires phosphorylation on two regulatory sites, Thr308 in the activation loop and Ser473 in the hydrophobic C-terminal regulatory domain. Moreover, phosphorylation at Ser473 appears to be critical for the regulation of AKT activity because selective dephosphorylation of Thr308 does not significantly affect the activity of AKT 48. As a serine/threonine protein kinase, AKT has been shown to suppress apoptosis by stimulating the activation of NF-κB 49,50. Nevertheless, the activation of ATM (Ser1981) instead of AKT was shown in the present work (Figure 3E-F). Furthermore, the activation of NF-κB was dependent on ATM since the phosphorylation of p65 decreased immediately upon treatment with an ATM inhibitor (Figure 3G). A previous study reported that in response to DNA damage, the activation of NF-κB was induced by the ATM-dependent phosphorylation of NEMO (the essential modulator of NF-κB), leading to the IKKβ mediated induction of the classical NF-κB pathway, which further supported our work 51. In addition, coimmunoprecipitation suggested an interaction among RMP, ATM and PARP, which could be further enhanced by RMP overexpression (Figure 3H). It has been reported that PARP senses stimuli and assembles IKKa, PIASy and ATM to form a signalosome upon DNA damage 52. Subsequently, IKKγ and small ubiquitin-like modifier (SUMO) are recruited, which in turn permits the activation of NF-κB 53. Based on the evidence, we suspected that the protein complex composed of RMP, ATM and PARP might mediate the tolerance to genotoxic agent CDDP (DNA damage inducer), thus inhibiting the intrinsic mitochondrial apoptotic pathway. Taken together, we demonstrated that RMP inhibits the intrinsic apoptosis induced by CDDP probably through ATM/NF-κB/Bcl-xl signaling pathway.

ATM is a key kinase to initiate DNA damage signaling cascade, which preferentially phosphorylates its target proteins at Thr-Gln and Ser-Gln motifs. As an important regulator of P53 upon DNA damage, ATM can not only directly phosphorylate p53 on Ser15, but also phosphorylates Chk2. Moreover, ATM impairs the activity of negative regulators of p53 such as the ubiquitin ligases cop1 and MDM2 54. Collectively, these alterations lead to the prompt accumulation of p53 and activation of its transcriptional functions. However, ATM is not the only regulator of P53 and in some cases the activation of P53 is independent of ATM 55.

As for the extrinsic apoptosis induced by TRAIL, PMP also turned out to play an inhibitory role. Concretely, the apoptotic rate of HCC exposed to 24h TRAIL treatment increased significantly after the depletion of RMP (Figure 5A-B). Moreover, the expression levels of representative pro-extrinsic apoptosis genes such as DR4 and caspase8 were also improved after the knockdown of RMP (Figure 5C-D). Notably, it has been reported that HCC cells acquired a resistant phenotype to TRAIL through the compensatory activation of NF-κB by facilitating the IKK-mediated IκB degradation and subsequent nuclear translocation of p65, leading to the up-regulation of many anti-apoptotic proteins 56,57. However, in the present work, our data showed that neither overexpression nor knockdown of RMP brought significant changes of P65 phosphorylation compared with control (Figure 6B). Notably, a previous study revealed that RMP could inhibit the expression of P53 through promoting the interaction between MDM2 and P53, thereby accelerating the ubiquitin-dependent degradation of P53, and mediating the modulation of autophagy in HCC cells 58. Based on this study, we suspected that RMP might control programmed cell death through P53 regulation. Here in this work, our data showed that the overexpression of RMP inhibited the expression of P53 in a concentration manner (Figure 7A-D). Moreover, the restoration of P53 expression significantly improved the apoptotic rate of RMP overexpressed HepG2 cells, which further confirmed our hypothesis (Figure 7E-F).

In conclusion, our observations not only highlight a novel exciting scenario and provide new formal evidence for a major role of RMP on apoptosis induced by chemotherapeutic agents, but also give prominence to the different signaling pathways through which RMP inhibits intrinsic and extrinsic apoptosis. These results shed light on novel strategies to overcome the drug-resistance issue in clinical cancer therapy.
